# Analysis of neural progenitors from embryogenesis to juvenile adult in *Xenopus laevis* reveals biphasic neurogenesis and continuous lengthening of the cell cycle

**DOI:** 10.1242/bio.013391

**Published:** 2015-11-30

**Authors:** Raphaël Thuret, Hélène Auger, Nancy Papalopulu

**Affiliations:** Faculty of Life Sciences, Michael Smith Building, University of Manchester, Oxford Road, Manchester M13 9PT, UK

**Keywords:** Birth dating, Cell cycle, Neural progenitor

## Abstract

*Xenopus laevis* is a prominent model system for studying neural development, but our understanding of the long-term temporal dynamics of neurogenesis remains incomplete. Here, we present the first continuous description of neurogenesis in *X.* *laevis*, covering the entire period of development from the specification of neural ectoderm during gastrulation to juvenile frog. We have used molecular markers to identify progenitors and neurons, short-term bromodeoxyuridine (BrdU) incorporation to map the generation of newborn neurons and dual pulse S-phase labelling to characterise changes in their cell cycle length. Our study revealed the persistence of Sox3-positive progenitor cells from the earliest stages of neural development through to the juvenile adult. Two periods of intense neuronal generation were observed, confirming the existence of primary and secondary waves of neurogenesis, punctuated by a period of quiescence before metamorphosis and culminating in another period of quiescence in the young adult. Analysis of multiple parameters indicates that neural progenitors alternate between global phases of differentiation and amplification and that, regardless of their behaviour, their cell cycle lengthens monotonically during development, at least at the population level.

## Abstract (precis)

**Summary:** Our work represents the first continuous analysis of neural progenitor behaviour in *X. laevis* from early embryogenesis to juvenile stage and identifies two phases of neurogenesis interrupted by a phase of slow increase of the progenitor pool.

## INTRODUCTION

Neurogenesis refers to the generation of post-mitotic neurons and glia from dividing progenitor cells, and it is a process necessary for the establishment of a functional central nervous system (CNS). Neurogenesis starts very early in the formation of an organism and although the exact phylotypic stage may vary between species, the first neurons generally appear after gastrulation at, or around, the time of neural tube closure. A large part of neurogenesis takes place during embryogenesis but it continues in post-natal stages. In many organisms, including mammals, it continues in the adult, restricted to a few areas of the CNS (D'Amico et al., 2011; for review see Kaslin et al., 2008; Schmidt et al., 2013), where cells are mostly quiescent but can be reactivated (Goldshmit et al., 2012; Kroehne et al., 2011; Muñoz et al., 2015; Zupanc and Ott, 1999). Thus, neurogenesis is a prolonged process. However, there is evidence from many organisms that neurogenesis can be divided in more or less distinct phases, characterised by the behaviour of neural progenitor cells (defined here in a broad sense as proliferating cells which may undergo fate determining symmetric or asymmetric divisions). For example, a change in behaviour over developmental time can be observed during the development of the mammalian cortex, where the properties of neural progenitor cells change in terms of marker gene expression, mode of division and generation of distinct neuronal subtypes (reviewed in Merkle and Alvarez-Buylla, 2006). Thus, during development neural progenitors may alternate between periods of expansion and periods of neurogenesis.

Early neurogenesis in lower vertebrates has been an invaluable model system in understanding the molecular control of neurogenesis, aided by the accessibility of the early embryo. For example, the *Xenopus* embryo has been instrumental in studying neurogenesis at the neural plate stage. Neural inducers (Piccolo et al., 1996; Zimmerman et al., 1996), Notch-Delta mediated lateral inhibition (Chitnis et al., 1995), and vertebrate proneural genes (Lee et al., 1995; Ma et al., 1996) were first described in this species. The early zebrafish embryo has also been instrumental and recent work in this model has been invaluable in understanding the molecular control of adult neurogenesis (Adolf et al., 2006; Chapouton et al., 2006, 2010; März et al., 2010; Rothenaigner et al., 2011).

The recent development and adaptation of better genetic manipulation tools in *Xenopus* open up an opportunity to study neurogenesis beyond the early embryonic stages and as a continuous process. Indeed, *Xenopus* with its distinct embryonic/larval and juvenile stages, punctuated by the process of metamorphosis offers a tremendous opportunity to study how transitions between different phases of neurogenesis are controlled in vertebrates.

As a first step in this direction, a thorough understanding of the neurogenic phases from early to late developmental stages is necessary. It is widely believed that two distinct phases of neurogenesis (primary and secondary) exist. It is thought that the primary phase establishes the embryonic CNS and a secondary phase generates the adult nervous system by largely replacing the primary nervous system (Hughes, 1957; Lamborghini, 1987). However, the evidence of distinct phases is fragmented because it is collated from different studies (Schlosser et al., 2002). Moreover, the data are often indirect and the temporal borders unclear as previous studies relied on identifying neurons well after their birth date, by the onset of late differentiation markers (Hartenstein, 1993; Lamborghini, 1980; Thors et al., 1982a,b).

Here, we describe for the first time in a single study how neural progenitors proliferate, self-renew and give rise to neurons over the whole course of embryonic, larval and post-metamorphic development. We have used Sox3, as a molecular marker of neural progenitors (for review see Pevny and Placzek, 2005) and combined it with cell proliferation reagents [phosphoHistoneH3 (pH3), BrdU] and a pan-neuronal marker (xMyT1) (Bellefroid et al., 1996; Bonev et al., 2011; Hudson et al., 2011), to obtain accurate snapshots of neural progenitor behaviour and neuronal birth rate over this extensive time course. Our observations show that the neural progenitor population is globally increasing during development and is maintained at least as far as the young adult frog. Two phases of intense progenitor division coincide with high neuronal production and are interrupted by a long period of apparent quiescence, enabling neural progenitors to slowly expand. Finally, the cell cycle length is gradually increasing to reach a maximum duration of 40 h at larval stages.

In conclusion, our work provides a dynamic cellular description of neural progenitor behaviour during the whole course of *Xenopus* development and lays the foundation for future molecular studies.

## RESULTS

### Sox3+ neural progenitors are maintained throughout *Xenopus* life

**Fig. 1.**
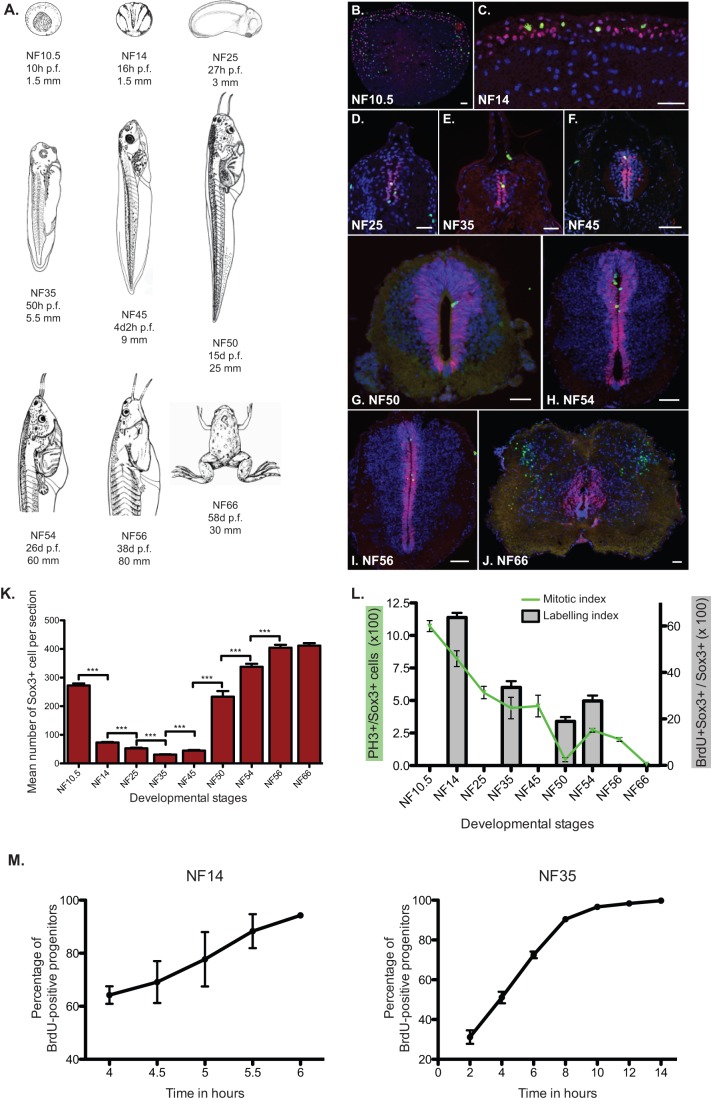
**Sox3+ progenitors are maintained across *Xenopus* development.** (A) Representation of *Xenopus laevis* stages used for the study. For each NF stage, the age in hours or days post-fertilisation (hpf or dpf) and the size of the animal (in mm) are provided. (B-J) Fluorescent immunodetection of Sox3 (red) and pH3 (green) at representative stages of *Xenopus* development. NF stages are indicated on each panel. Nuclei are counterstained with DAPI. Scale bars=50 µm. Note that during metamorphic stages, we identify a population of cells negative for Sox3 outside the ventricular zone but positive for PH3 in the posterior hindbrain/anterior spinal cord. These cells could represent a population of non-neurogenic glia such as astrocytes or oligodendrocytes (Maier and Miller, 1995; Yoshida et al., 1999). (K) Mean absolute number of Sox3-positive cells per section during development. After the restriction of the expression of Sox3 to the neural plate, the number of positive cells decreases slightly until NF25 to augment and then reach a plateau by NF54. (L) Quantification of mitotic index (green curve) and labelling index (grey bars) of Sox3+ progenitors. The number of pH3-positive nuclei amongst Sox3-positive ones has been counted. Mitotic index is found to be high during neurulation (NF 14 to 25) and prometamorphosis (NF54-56). In between NF25 and NF54, the mitotic index decrease gradually to become nearly negligible at NF50. The labelling index represent the percentage of BrdU-positive Sox3-positive cells from animals injected with BrdU and fixed 3 h later. (M) Cumulative BrdU incorporation in neural progenitors at NF14 and NF35. More than 95% of the Sox3+ progenitors are positive for BrdU after 6 h at NF14 and after 10 to 12 h at NF35. Data represented as mean±s.e.m. ***P<0.001.

We studied a long period of development, covering almost 2 months (Fig. 1A, Fig. S1D) starting from mid-gastrula [Nieuwkoop and Faber stage (NF)10.5] to juvenile stage (NF66, Fig. 1A). Larval stages were chosen on the basis of a screen using pH3 antibody and looking for changes of mitotic activity on whole mount dissected CNS (data not shown). To facilitate comparison between developmental stages, we focused on one area of the CNS. We chose the anterior spinal cord/posterior hindbrain because it was reliably identifiable by morphological landmarks (position of the otic vesicle, tapering of hindbrain roof) during the whole course of development after neural tube closure.

We analysed the immunoreactivity against Sox3 at mid-gastrula stage (NF10.5), neural plate (NF14), early tadpole (NF25), mid-tadpole (NF35), late tadpole (NF45), pre-metamorphic stage (NF50), metamorphic (NF54-56), and post-metamorphic juvenile stage (NF66; Fig. 1B-J). Prior to neural induction, Sox3 is expressed in all ectodermal cells of the gastrula (NF10.5, Fig. 1B) (Penzel et al., 1997; Zhang et al., 2003). From neural induction onwards, Sox3 becomes restricted and maintained to neural progenitors (Rogers et al., 2008). Analysis of pH3 labelling revealed that all mitotic cells are contained within the Sox3 domain (with the exception of some cells located at the lateral margins of the neural tube at NF66, Fig. 1B-J). Moreover the domain of expression of Sox3 does not overlap with immunoreactivity with neuronal markers such as xMyT1 (Fig. S1B). Sox3 expression appears heterogeneous in intensity at the single cell level, therefore, it is likely that Sox3 labels neural progenitors, regardless of the possible existence of different subtypes or dynamic expression.

The data revealed that Sox3+ cells changes organization over the different developmental stages (Fig. 1B-J). At the neural plate stage (NF14), Sox3+ cells are organised into two neuroepithelial layers, a deep and a superficial one, as previously described (Schroeder, 1970, Fig. 1C). At neural tube closure (NF25, Fig. 1D), deep and superficial cells intercalate (Davidson and Keller, 1999) resulting in Sox3+ cells lining up the ventricle in a one-cell width layer (ventricular zone). This monolayer structure is maintained to early tadpole (NF35, Fig. 1E) but changes into a 2 to 4-cell width zone around the lumen of the spinal cord gradually in the stages leading to metamorphosis (from NF35 to NF50, Fig. 1E-G). During metamorphosis (NF54 to NF56, Fig. 1H-I), the progenitor zone thins again, although does not quite become a monolayer. After the climax of metamorphosis (NF66), the cytological structure of the spinal cord changes again compared to NF56, as it has been previously described (Thors et al., 1982b) (compare Fig. 1J to I). The length of the lumen of the spinal cord is reduced from approximately 800 µm to 400 µm. Sox3+ progenitors are redistributed in to a wider area around the ventricle, perhaps forming a ventricular and a subventricular zone. Overall, the cell density seems to be reduced in both the progenitors and the neurons compartments with a massive expansion of the white matter. Dividing cells (PH3+) outside the ventricular and subventricular zones appear towards the margins of the neural tube, coincident with the appearance of white matter.

The number of progenitor cells (number of Sox3+ cells per section, Fig. 1K and Table in Fig. S1D) also changes during development. A sharp decrease is observed between gastrula (NF10.5) and neurulation (NF14); however, this is most likely due to neural induction that restricts the expression of Sox3 to neural progenitors. Between NF14 and 35, the number of Sox3+ cells decreases progressively from a mean of 70 to 30 Sox3+ cells per section (Fig. S1D). Some of this may be attributed to the elongation of the embryo between NF25 and NF35 (the total size of the embryo changes from 3 to 6 mm). A 1.5-fold increase is observed between NF35 and NF45 and a 6-fold increase between NF45 and NF50. This massive expansion may be responsible for the thickening of the progenitor zone at NF50 (Fig. 1G-I). In metamorphic stages (between NF50 and 56), the number of Sox3+ nuclei rises less sharply but steadily, reaches its maximum by NF56 and is maintained at NF66.

### Sox3+ cell division rate peaks during neurulation and pro-metamorphosis

To determine the rate of division of neural progenitors during development, we quantified the mitotic index, defined as the number of Sox3+ nuclei that were positive for the mitotic marker pH3 at any one time over the total number of Sox3+ progenitors (Fig. 1B-J and green line in L). This index enables us to observe if the population of neural progenitors is actively dividing at a particular stage of development and gives an indication of cell cycle length; assuming a constant length of M phase, a decline in the proportion of progenitors that are in M-phase shows that M-phase occupies a smaller portion of the cell cycle, indicative of lengthening of the cell cycle.

We observed that during gastrulation (NF10.5), about 10% of the cells in the ectoderm are found in mitosis. At early neurula (NF14), this percentage drops to 7.5% in the neuro-ectoderm and then decreases further to 5% at early tailbud stage (NF25). The mitotic index remains at this basal level of 5% up to larval stage (NF45). The biggest decrease is observed at pre-metamorphosis (NF50), when the mitotic index falls to just 0.4%, suggesting a really long cell cycle or a period of quiescence. Mitotic activity peaks again transiently during metamorphosis (NF54 and 56) to finally fade at juvenile stage (NF66).

We next estimated the labelling index on stages of development that are representative of the changes of the mitotic index to confirm this result by an independent method (Fig. 1L, charts bars). The labelling index is the quantification of BrdU+ cells amongst the Sox3+ population after a short pulse of BrdU (3 h). For all the stages analysed, the labelling index is in accordance with the mitotic index. At NF14, when 7.5% of the cells are found in mitosis, around 60% of the cells have incorporated BrdU indicative of fast cycling progenitors. At NF35, only 33% of the progenitors are positive for BrdU concomitant with the diminution of the mitotic index. At NF50, when the mitotic index in very low, only 19% of the progenitors are positive for BrdU and this proportion rises again to 28% at NF54.

Cumulative EdU incorporation of Sox3+ progenitors at NF14 and NF35 reveals that almost the entire Sox3+ population can be labelled within 6 to 12 h (depending on the stage; see Fig. 1M) confirming that Sox3-positive progenitors are actively dividing. However, Sox3 continues to be expressed in progenitors even at stages when very few cells in mitosis are observed; therefore, Sox3 seems to label both actively (rapidly) dividing and quiescent (or slowly dividing) progenitors.

This data indicate that neural progenitors display two periods of active cell division, albeit with different cell cycle lengths (see also later); one from neurulation (around NF14) to larval stage (NF45) and a second one at early metamorphic stages (NF54 and 56). Neural progenitors also display two periods of relative quiescence, one before metamorphosis (NF50) and one when metamorphosis is complete (NF66). Notably, the distribution of mitotic cells at NF54 and NF56 seems different between the dorsal and the ventral part, suggesting some heterogeneity in progenitor behaviour at these stages (Fig. 1H,I).

### Progenitors alternate between neurogenesis and expansion

**Fig. 2.**
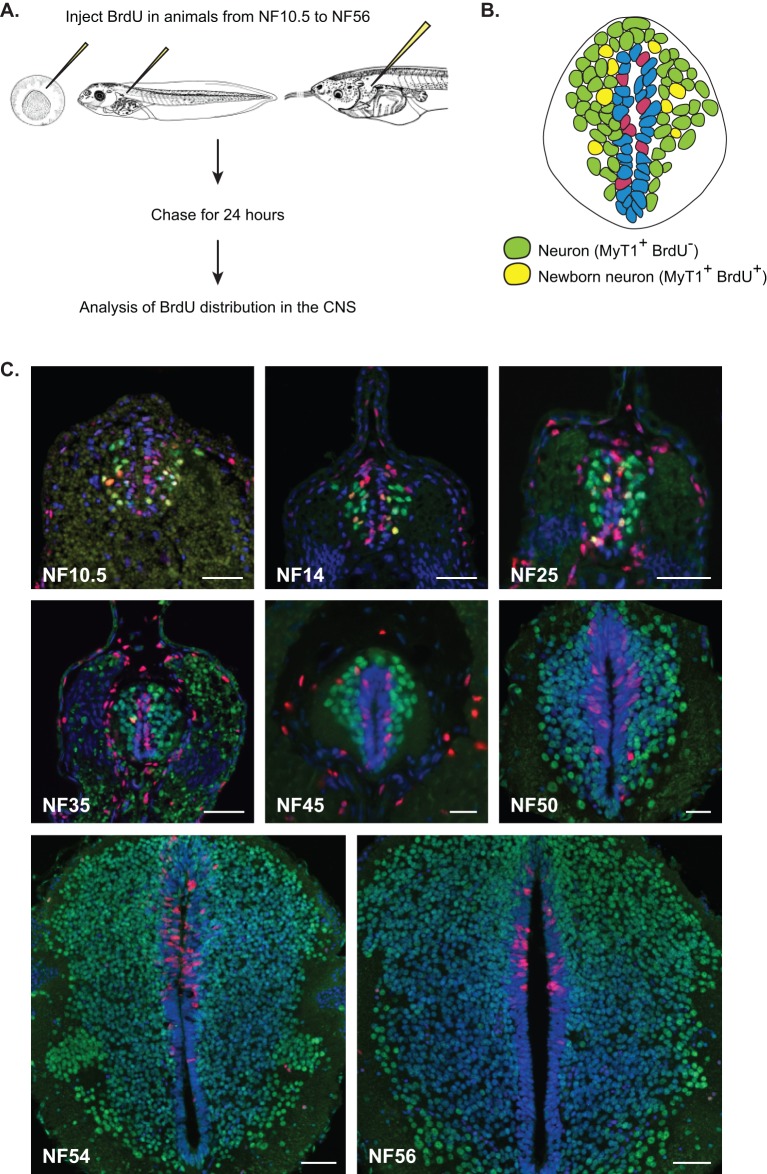
**Neuronal birth during development.** (A) Rationale of the birth dating experiment. Animals are injected with BrdU and kept for 24 h to allow BrdU incorporation and cell division to occur. (B) Schematic representation of spinal cord with identified cell populations. In a section of spinal cord, we can identify two categories of cells. (C) Fluorescent immunostaining against xMyT1 (green) and BrdU (red). Nuclei were counterstained with DAPI. Each developmental stage is indicated on the images. Scale bars=50 µm.

In order to understand the balance between neuronal production and progenitor expansion across development we looked both at the expansion of the neuronal population (neuronal birth) and the fate of dividing progenitors. We injected BrdU at various developmental stages ranging from NF10.5 to NF56 (Fig. 2A) and analysed BrdU incorporation 24 h later. In spite of the long time prior to analysis, BrdU is only available for 2.5 h (see Materials and Methods) therefore we were able to track progenitors that were in S-phase for the short period that BrdU was available and to identify them or their progeny 24 h later. BrdU persists at detectable levels in daughter cells after up to four divisions (Kiel et al., 2007). To assess the generation of neurons we used a pan-neuronal nuclear antibody against xMyT1, a transcription factor expressed from the early steps of neuronal differentiation pathway (Bellefroid et al., 1996). xMyt1 immunostaining is not detected either in progenitor cells that are Sox3-positive nor in neuronal precursors/prospective neurons that express X-Delta-1 (see Fig. S1B,C) (Chitnis et al., 1995; Henrique et al., 1995). The location of xMyt1 cells is more lateral than X-Delta-1 (see Fig. S1C) (Bellefroid et al., 1996; Schlosser et al., 2002), suggesting that xMyt1 is a slightly later marker and a good post-mitotic pan-neuronal marker.

Two different categories of neurons were identified according to their immunoreactivity (Fig. 2B). (1) Neurons that were already differentiated at the time of labelling (BrdU− xMyT1+, Fig. 2B green nuclei). (2) Neurons that were born during the period of labelling from dividing progenitors (BrdU+ xMyT1+, Fig. 2B yellow nuclei).

**Fig. 3.**
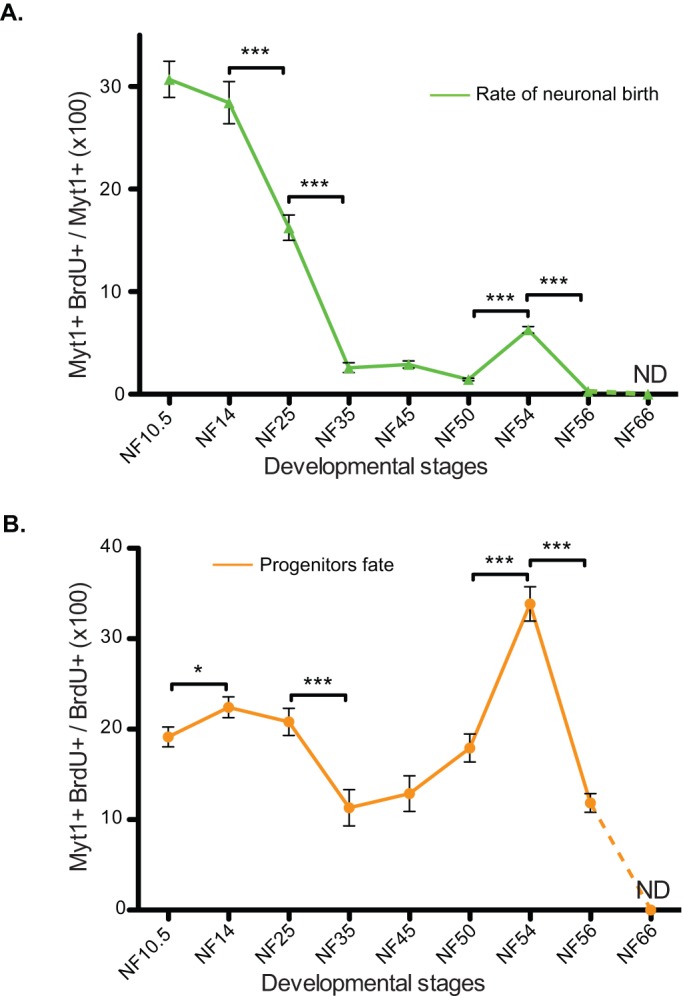
**The behaviour of neural progenitors changes during development.** The quantification of the birth dating results enables the estimation of two parameters according to the populations considered. Since mitotic index was negligible at NF66, no birth dating data have been collected for this stage and values have been extrapolated to 0 (dashed lines). (A) Rate of neuronal birth. This graph represents the percentage of BrdU-positive cells among the xMyT1-positive cells and corresponds to the proportion of newly born neurons appeared during the last 24 h. Two main phases of neuronal birth can be identified at NF14 and NF54. In between, the rate of newly born neurons is very low. After NF54, the rate of newly born neurons decreases again. (B) Progenitors fate. This diagram represents the percentage of xMyT1-positive cells among the BrdU-positive population and is an indication of the choice made by progenitors to stay in the cell cycle by proliferating or to exit the cell cycle by differentiating into neurons. Data represented as mean±s.e.m. ***P<0.001, *P<0.05.

We determined the rate of neuronal production, by quantifying the proportion of newly born neurons (BrdU+ MytT1+) during the time of labelling over the entire neuronal population (all MyT1+ nuclei) at the time of analysis (category 2 over 1+2, see above; Fig. 3A). We observed two main waves of neuronal birth. The first wave occurs during neurulation (primary neurogenesis, peaking at NF14) where 30% of the neurons found 24 h later in the spinal cord were born during this time period. The rate of neuronal birth rapidly decreases two-fold at NF25 to reach a low, basal level of neurogenesis of about 2% of newly born neurons in 24 h at NF35. This minimal level of neuronal birth is maintained throughout the end of embryogenesis up to pre-metamorphic stages. The second peak of neurogenesis is then happening during metamorphosis, at NF54 (Fig. 3A), where about 5% of the neuronal population is positive for BrdU, demonstrating their birth during the last 24 h. As evidenced by the mitotic index, most of the BrdU incorporation is observed in the dorsal part of the spinal at NF54 and 56, suggesting that Sox3+ progenitors are heterogeneous at these stages.

The analysis above estimates how many new neurons are added to the neuronal pool at any given point, but to gain insight into progenitor behaviour, we next looked at the outcome of progenitor division. We quantified the proportion of BrdU-positive cells that differentiated at different stages (xMyT1+ and BrdU+ over all BrdU+ cells, Fig. 3B). This shows that progenitors do not give rise to a homogeneously distributed progeny over development. On the contrary, we observe several changes in cell fate decision during the period studied. First, during early embryogenesis (from NF10.5 to NF 25), 20% of the BrdU- incorporating cells are becoming neurons. Between NF35 and 50, only 10% of BrdU+ cells become neurons (Fig. 3B), consistent with a low level of neuronal production (Fig. 3A). During this time, even though the mitotic and labelling index are steadily declining (Fig. 1L), the neural progenitors increase their number (Fig. 1K) more than giving rise to neurons (Fig. 3B), although a low level neuronal production persists (Fig. 3A). Finally, at metamorphosis, when neuronal production peaks again at NF54 (Fig. 3A), we observe that 30% of the cells incorporating BrdU give rise to neurons (Fig. 3B). Since the number of progenitor cells does not increase at this stage (Fig. 1K), we suggest that we are witnessing a switch in progenitor behaviour from self-renewal towards neuronal production.

### The cell cycle length gradually increases during *Xenopus* development

**Fig. 4.**
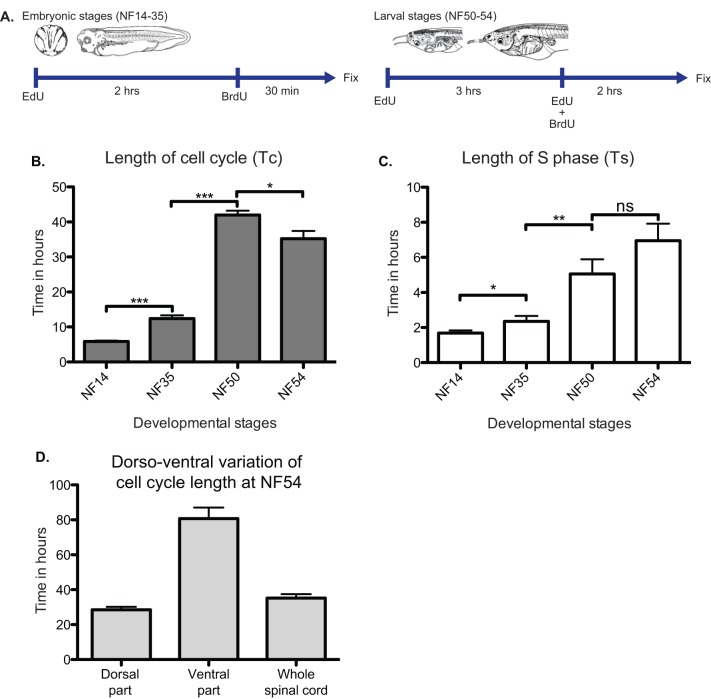
**Estimation of cell cycle length during *Xenopus* development.** (A) Protocols used for estimation of cell cycle length by dual pulse S-phase labelling. Different strategies have been used for stages corresponding to primary neurogenesis (NF14, *n*=13), progenitor amplification phase (NF35, *n*=9), prometamorphosis (NF50, *n*=5), and secondary neurogenesis during metamorphosis (NF54, *n*=5). (B) Total cell cycle length (Tc) and (C) S-phase length (Ts) at corresponding stages. Both Ts and Tc are increasing during development in a manner that seems independent of the neural progenitors behaviour. (D) Cell cycle length of the dorsal part versus the ventral part of the spinal cord at NF54. The overall cell cycle at this stage is 35 h represent a composite of a heterogeneous population of Sox3 progenitors where dorsal progenitors cycle faster (25 h) than the ventral ones (80 h). Data represented as mean±s.e.m. ***P<0.001, **P<0.01, *P<0.05.

Previous studies have shown that progenitors terminally dividing to give rise to neurons have a longer cell cycle (most particularly G1 phase) than progenitors that self-renew (Calegari, 2005; Salomoni and Calegari, 2010), although the S-phase is shorter (Arai et al., 2011; Cabochette et al., 2015). Since the majority of neuronal production in *Xenopus* takes place in two developmental phases, we wondered if this was linked to distinct changes of the cell cycle. In order to estimate the cell cycle length, we used the method of dual pulse S-phase labelling combining EdU, BrdU as well as Sox3 as a marker of the growth fraction (Martynoga et al., 2005; Peco et al., 2012; Sabherwal et al., 2014). We assumed that the growth fraction was identifiable to the Sox3+ progenitor population according to our cumulative BrdU incorporation results (Fig. 1M). We applied this method, during the first period of neuronal generation (neurulation, NF14 and mid-embryogenesis, NF35), the period of pre-metamorphic quiescence (larval stage, NF50) and the second period of neuronal generation (metamorphosis, NF54), adapting the protocol in a stage specific manner (Fig. 4A). This enabled us to determine the length of S-phase and the total duration of the cell cycle in the population of Sox3+ progenitors at these developmental stages (Fig. 4B,C). Our results show that the cell cycle gradually increases in length over developmental time (Fig. 4B). The average cell cycle is 5 h 52 m long at NF14, doubles in length at NF35 (12 h 24 m) and then increases again to a duration of 42 h at NF50. At NF54, the cell cycle shortens to a duration of 35 h, indicative of a reactivation of neural progenitors to produce new neurons. The same kind of trend is seen regarding the S-phase duration (Fig. 4C). We estimate that S-phase last for about 1 h 40 m at neural plate stage (NF14) and 2 h 20 m at mid-embryonic stage (NF35). Then, S-phase reaches 5 h at NF50 and remains of a comparable duration at NF54 (6 h 57 m). The lengthening of the cell cycle is consistent with the gradual decrease in the mitotic index between NF14 and NF50 (Fig. 1K). We subsequently observe a shortening of the cell cycle at NF54 in accordance with the increase of the mitotic index identified at this stage. This seems mostly due to the dorsal part of the spinal cord that is cycling much faster than the ventral part (Fig. 4D), confirming our observations of the distribution of aPH3 and BrdU staining at this stage (Fig. 1I, Fig. 2C). Our data also confirm and extend the conclusions of Frederick and Andrews that cell cycle is elongating, mostly by an increase of the G1-phase, which were based on flow cytometry of isolated nuclei from whole developing *Xenopus* embryos (Frederick and Andrews, 1994).

## DISCUSSION

We have presented a continuous cellular description of neurogenesis as a necessary first step to allow cellular behaviour to be understood from a molecular, mechanistic point of view. Our study revealed two distinct phases of neurogenesis, which is consistent with the previous view of primary and secondary neurogenesis based on compilation of cellular studies (Schlosser et al., 2002) or genes expression pattern of proneural genes in zebrafish and *Xenopus* (Mueller and Wullimann, 2003; Wullimann et al., 2005).

Summarising our data, we conclude that primary neurogenesis, corresponds to an initial neurogenic wave that takes place at neurulation (NF14), gradually declines until the mid-tadpole stage (NF35), and stays at a very low level until metamorphosis (NF54) when a second peak of neurogenesis is observed. During the second peak of neurogenesis, a smaller percentage of new neurons are added to the existing neuronal pool compared to the first (Fig. 3B). However, from the progenitors' point of view, secondary neurogenesis is a significant event; over 30% of progenitors present in the metamorphic neural tube (NF54, Fig. 3C) engage in neurogenic divisions, while only approx. 22% do so during primary neurogenesis (Fig. 3C). To reconcile the highly neurogenic progenitor fate (Fig. 3C) with the lower rate of generation of new neurons at NF54 (Fig. 3B), one has to consider that new neurons are added to a much larger population of existing neurons in NF54 than NF14.

Our data also revealed two phases of quiescence and allowed us to map these precisely on a temporal scale. The first period of quiescence takes place before metamorphosis (NF50), when we observed a very low number of cells in mitosis (pH3, Fig. 1L) as well as a very low rate of neuronal birth (Fig. 3A). The fact that there is a low but residual BrdU incorporation in the first period of quiescence indicates that some progenitors may cycle very slowly at NF50. Consistent with this, we showed a dramatic lengthening of the average cell cycle between NF35 to NF50 by direct measurements. Slow cycling is perhaps what one would expect if during the first period of quiescence progenitor cells are poised for reactivation at metamorphosis (NF54). Despite the maintenance of the expression of Sox3 in cells around the ventricle, the second period of quiescence observed in the post-metamorphic animal seems more definitive as the mitotic index falls to 0.1% between NF56 and NF66, consistent with other reports (D'Amico et al., 2011). It is also observed in other adult organisms, including mouse (Altman, 1969a,b; Grandel et al., 2006). We propose here the terms primary and secondary quiescence to describe these distinct events, in line with the terms primary and secondary neurogenesis. Both periods of quiescence are interesting in that they provide a reservoir of potentially re-activatable stem cells. Exit from secondary quiescence may be of particular interest in adult regeneration studies while exit from primary quiescence may be of particular interest in understanding the hormonal control of neuronal progenitor reactivation. Primary quiescence in *Xenopus* may indeed be a good model to study hormonally driven large-scale changes in neural progenitor behaviour in a vertebrate. In this perspective, our findings suggest similarities to the *Drosophila* model where it has been shown that neuroblasts enter quiescence during late embryogenesis, creating a break between embryonic and post-embryonic neurogenesis. Post-embryonic neuroblasts are then reactivated by insulin/IGF pathway (Chell and Brand, 2010). It is interesting to note that NF35 marks the onset of a period of change in the behaviour of progenitors, as evidenced by the elongation of the cell cycle length (also witnessed by the changes in mitotic and labelling indices) and rate of neuronal birth which intensifies at NF45 and lasts until NF50. At NF35, thyroid hormone receptors (TR) start to be expressed (Eliceiri and Brown, 1994) and act as repressors in the absence of thyroid hormone (TH). At NF54, TH starts to be produced (Leloup and Buscaglia, 1977) and binds to TR, converting repressive unbound TR to activating bound TR, enabling the metamorphic process. These changes in TR activity could be partially responsible for the change in progenitor behaviour. Notably, it has been shown before that TH treatment had an effect on neural progenitors, modifying their cycling properties (Denver et al., 2009).

By integrating information obtained by different methods, we also gained some insight into the behaviour of progenitors through developmental time, which would not have been possible with any of these methods alone. In principle, neural progenitors may divide symmetrically in terms of fate to produce 2 more progenitors (symmetric proliferative division) or 2 neurons (symmetric terminal division). Alternatively, they may also divide asymmetrically in terms of fate, to produce one neuron and one progenitor (asymmetric division). Of those, only symmetric proliferative divisions increase the number of progenitor cells, while terminal symmetric divisions decrease it and asymmetric divisions maintain it. Thus, the change in the number of progenitor cells (Fig. 1K) taken together with the rate of neuronal birth (Fig. 3A) and the fate of progenitor divisions (Fig. 3B) gave us a clue as to the mode of their division.

First, during early embryogenesis (from NF10.5 to NF 25), 20% of the BrdU incorporating cells are becoming neurons (Fig. 3B), which when taken together with the approximate stable number of progenitors between NF14 and 25 indicates that, at these stages, differentiated neurons are generated without depleting the progenitor pool, indicating asymmetric divisions. From neurula (NF14) to tadpole stage (NF35) the number of progenitors per section decreases by half. The most likely explanation to the reduction of progenitor numbers between these stages would be the antero-posterior elongation of the embryo, doubling its size from NF14 to NF25 (15 to 30 mm; Nieuwkoop and Faber, 1956). There is also an intense generation of neurons at this period; in fact, a higher percentage of neurons (30%) are generated in the 24 h following neurulation than at any other time in the formation of the animal (see Fig. 3A). Thus, we suggest that the data are consistent with an asymmetric neurogenic mode of division for the majority of progenitors at this stage, as this would tend to conserve rather than drastically reduce the number of progenitors, while allowing neuronal birth.

From NF35 to NF50, the rate of neuronal birth is very low and the number of progenitors increases, implying symmetric proliferative divisions between these stages. The massive increase of progenitor number observed between NF45 and NF 50 (Fig. 1K) may seem paradoxical if one considers the low level of BrdU incorporation at NF 45 and NF50 i.e. the stages preceding primary quiescence. However, it can be explained if one considers that cell division is integrated over a long period of time; for example, there is 2 days between NF35 to NF45 and 11 days between NF45 to NF 50 (see Fig. 1A). Alternatively, there may be a peak in proliferation, which takes place in a narrow window between NF45 and NF50 that we did not detect. Nonetheless, the fact that some divisions are neurogenic in fate (Fig. 3B) suggests that between these stages there is a mixture of behaviour where the majority of divisions are (slow) symmetric proliferative but some are asymmetric neurogenic.

Subsequently, during the period of secondary neurogenesis, which spans metamorphosis (between stages NF50 and NF56), the rate of progenitor pool expansion slows down and neuronal production picks up, implying a switch to an asymmetric neurogenic mode of division. The number of Sox3-positive progenitor cells stabilises between NF56 and NF 66 (Fig. 1J) and we no longer observed any mitotic cells. Of course, at all stages, the same outcome could be explained by more complicated scenarios, if there is a heterogeneity of behaviour in the progenitor pool. Nevertheless our interpretation of the data form a useful preliminary hypothesis that remains to be tested by more sophisticated methods.

Finally, our data revealed that neural progenitors display a seven-fold change in cell cycle length from neural plate to metamorphic stages in an apparently gradual increase. This was observed indirectly by the decrease in the mitotic index (Fig. 2L) and directly, by measurement of cell cycle and S phase length (Fig. 4). The short cell cycle length (6 h) observed during primary neurogenesis is similar with that observed, in the *Xenopus* retina (El Yakoubi et al., 2012), and is conserved at comparable development stages in zebrafish (He et al., 2012; Leung et al., 2011; Lyons, 2003), chicken (Peco et al., 2012) and mouse (Arai et al., 2011; Calegari, 2005; Caviness et al., 1995). In the mouse cortex, the transition from neuro-epithelial cells to progenitors to slow cycling stem cells during development have been shown to be accompanied by a gradual slowing down of the cell cycle to finally reach quiescence where cells can be reactivated (Lange and Calegari, 2010). Indeed, in our system, cell cycle gradually lengthens to reach a maximum length of about 40 h at NF50. At NF54, during secondary neurogenesis, we observe a shortening of the cell cycle, mainly coming from the dorsal part of the spinal cord indicative of the reactivation of neural progenitors and their subsequent differentiation.

In conclusion, our comprehensive analysis has revealed persistence of stem cells and population level changes during development. Specifically, there are two periods of neurogenesis and two periods of quiescence, accompanied by a continuous lengthening of the cell cycle and a possible alternation in division mode. These conclusions do not exclude the possibility of heterogeneity in progenitor behaviour (suggested by expression of TRα and TRβ; Denver et al., 2009) and remain to be confirmed by single cell analysis. In future, more comprehensive analyses at the single cell level will be needed to gain further insight into neural progenitor behaviour at different stages of development.

## MATERIALS AND METHODS

### Embryos, tadpoles and developmental stages choice

Animal experiments were approved by the University of Manchester Ethical Review Panel and were undertaken under UK Home Office project license PPL 70/7648. *Xenopus laevis* females were purchased from the *Xenopus* stock centre, Portsmouth, UK. Embryos were obtained by *in vitro* fertilization of hormone-induced laid eggs. Developmental stages were determined and are referenced in the text according to Nieuwkoop and Faber *Xenopus* table of development (Nieuwkoop and Faber, 1956). Once the animals reached feeding stage (NF45), they were kept at 16°C in filtered water and fed daily with spirulina until they reach the required NF stage for experimentations. The time interval between different stages is shown in Fig. 1A and recapitulated in Fig. S1D.

### Bioavailability of uridine analogues

In order to set up our experiments, we first determined the time a uridine analogue was available for labelling after injection (Fig. S1A). To estimate this parameter, we performed a dual pulse S-phase labelling at NF35 using BrdU and 5-Ethynyl Uridine (EdU). After an initial injection of BrdU (10 mM, Roche), embryos were kept for an increasing amount of time (from 1 to 3.5 h) before being injected with EdU (10 mM, Life Technologies) and finally fixed 30 min later. The minimum amount of time necessary to see the appearance of EdU+ only cells corresponds to the time where BrdU is no longer available. We thus estimated that BrdU is available for a period of time ranging between 2 and 2.5 h (Fig. S1A) since EdU+ only cells starts to be identified by 2.5 h (arrowheads) and become more numerous afterwards.

### Birth dating experiments

In order to analyse the fate of dividing progenitors at different stages of development, we used the method of birth dating consisting of following the progeny of cells that incorporated a S-phase marker such as BrdU. Animals were injected with BrdU (10 mM, Roche) at the desired NF stage and kept for 24 h at 18°C (Fig. 2A) in order to allow neuronal differentiation to happen in the progeny of dividing cells. After fixation, samples were processed according to the method described in the immunofluorescence section. An additional incubation in 2 N HCl at 37°C for 30 min was performed before the blocking step of primary antibody incubation in order to enable accessibility to the BrdU antigen.

### Cumulative EdU incorporation

To estimate the growth fraction (i.e. percentage of cycling cells in the Sox3 population), we performed cumulative EdU incorporation at NF14 and NF35. Embryos were injected every 2 h with 10 nM EdU (4.2 nl at NF 14, 3×4.2 nl at NF35) and fixed every 30 min for NF14 or 2 h for NF35. Samples were then processed for Sox3 immunostaining and EdU detection and consecutively imaged. The percentage of Edu+Sox3+ was estimated from five consecutive sections taken from three different embryos for each time point.

### Cell cycle length analysis

Dual pulse S-phase labelling (Martynoga et al., 2005; Peco et al., 2012; Sabherwal et al., 2014) was used to estimate the length of the S-phase (Ts) and subsequently determine the cell cycle length (Tc) in the population of Sox3 progenitors. This method relies on the fact that the percentage of cells in a particular phase of the cell cycle is proportional to the length of this phase. The length of the S phase is estimated by two successive injections of different uridine analogues (in our case EdU and BrdU). Once the S phase length has been determined (see below), we can extrapolate the length of the whole cell cycle in the considered population. Practically, a first injection of EdU is provided and chased for a fixed amount of time (Texp) when BrdU is injected and chased for the minimal duration for reliable detection. Animals are then fixed and processed for EdU, BrdU and Sox3 detection. Three cell populations are identifiable:

1. Sox3+ cells corresponding the whole pool of cycling cells (proliferating fraction, P cells).
2. Sox3+ EdU+ only cells represent cells that left S phase before BrdU was available (leaving fraction, L cells).
3. Sox3+ EdU+ BrdU+ cells representing the cells that were in S phase at the time of fixation (replicative fraction, S cells).

According to Nowakowski (1989), the ratio of the duration of two phases of the cell cycle is equal to the ratio of the number cells in each of these phases. Hence, we can estimate the S phase length by the following relation:

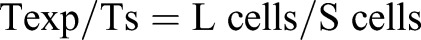


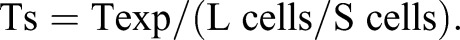


Similarly, we can determine Tc by using a similar formula:

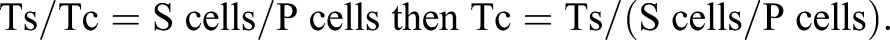
We estimated that uridine analogues detection was robust and clear after 30 min at NF 14 and NF35 and after 2 h at NF 50 and NF54. Animals were isolated at the desired NF stage and injected first with EdU (10 mM in DMSO, Life Technologies). After 2 to 3 h incubation (depending on the developmental stage, Fig. 4A), an injection of BrdU (10 mM in DMSO, Roche, or EdU+BrdU at 10 mM each in case of 3 h incubation) was provided. Animals were then sacrificed 30 min to 2 h later according to their stage and processed as described in the immunofluorescence section. EdU detection was made using Click-iT EdU imaging kit from Life Technologies as described in Auger et al. (2012). BrdU antigen retrieval using 2 N HCl was performed after EdU staining and before primary antibody blocking step. The different cells categories described earlier were then counted on 10 consecutive sections from at least 5 different animals.

### Immunofluorescence

Embryonic stages (up to NF45) were fixed in MEMFA for 1 h at room temperature, and then washed and kept in methanol at −20°C until embedding. Once anaesthetised in MS222, larval stages (from NF50) were fixed in 4% paraformaldehyde in 1× PBS overnight at 4°C. Central nervous systems were then dissected and kept in methanol at −20°C until further processing. Embedding, cryosectioning and immunofluorescence were performed as described in Auger et al. (2012). The following primary antibodies were used: rabbit anti-Sox3 (Eurogentec, custom made as described in Zhang et al., 2003, 1:1000), rabbit anti-xMyt1 (Eurogentec, custom made as described in Sabherwal et al., 2009, 1 in 1000), mouse anti-pH3 (Abcam Ab14955, 1 in 300), mouse anti-BrdU (Roche Cat. No. 11 170 376 001, 1:500) and mouse anti-BrdU MoBU (Life Technologies B35128, 1:300). Corresponding secondary antibodies coupled to Alexa fluorophores (anti-mouse Alexa 488, anti-mouse Alexa 568, anti-rabbit Alexa 488, anti-rabbit Alexa 568, anti-rabbit Alexa 647) were purchased from Life Technology and used at 1:500 dilution. Double immunostaining with same species antibody was performed using Zenon Kit (Life Technologies). Once a regular antibody staining was performed for one of the antibodies, the other antibody was complexed with the Zenon IgG and incubated on the sections overnight at 4°C. Immunofluorescence combined with *in situ* hybridisation was performed as described in Auger et al. (2012). X-Delta-1 is a kind gift from Eric Bellefroid (used in Bellefroid et al., 1996)

### Images collection and data analysis

Images were collected on an Olympus FV1000 confocal microscope or a Nikon Widefield microscope. For each quantified value, at least 3 different animals were sampled on 10 consecutive 12 µm sections. Quantification was then manually performed using the Cell Counter plugin of ImageJ. Data shown are expressed as the mean±s.e.m. and are presented in Fig. S1D. Normality tests were applied on datasets and appropriate *t*-tests were used in order to assess the statistical significance of the data. Significance values are ***P<0.001, **P<0.01, *P<0.05.
